# Etiology and Factors Associated with Pneumonia in Children under 5 Years of Age in Mali: A Prospective Case-Control Study

**DOI:** 10.1371/journal.pone.0145447

**Published:** 2015-12-22

**Authors:** Thomas Bénet, Mariam Sylla, Mélina Messaoudi, Valentina Sánchez Picot, Jean-Noël Telles, Abdoul-Aziz Diakite, Florence Komurian-Pradel, Hubert Endtz, Souleymane Diallo, Gláucia Paranhos-Baccalà, Philippe Vanhems

**Affiliations:** 1 Infection Control and Epidemiology Unit, Edouard Herriot Hospital, Hospices Civils de Lyon, Lyon, France; 2 University of Lyon 1, Lyon, France; 3 International Center for Infectiology Research (CIRI), Lyon, France; 4 Pediatrics department, Gabriel Touré Hospital, Bamako, Mali; 5 Emerging Pathogens Laboratory, Fondation Mérieux, Lyon, France; 6 Department of Medical Microbiology and Infectious Diseases, Erasmus MC, Rotterdam, The Netherlands; 7 Centre d'Infectiologie Charles Mérieux, Bamako, Mali; Kliniken der Stadt Köln gGmbH, GERMANY

## Abstract

**Background:**

There are very limited data on children with pneumonia in Mali. The objective was to assess the etiology and factors associated with community-acquired pneumonia in hospitalized children <5 years of age in Mali.

**Methods:**

A prospective hospital-based case-control study was implemented in the Pediatric department of Gabriel Touré University Hospital at Bamako, Mali, between July 2011-December 2012. Cases were children with radiologically-confirmed pneumonia; Controls were hospitalized children without respiratory features, matched for age and period. Respiratory specimens, were collected to identify 19 viruses and 5 bacteria. Whole blood was collected from cases only. Factors associated with pneumonia were assessed by multivariate logistic regression.

**Results:**

Overall, 118 cases and 98 controls were analyzed; 44.1% were female, median age was 11 months. Among pneumonia cases, 30.5% were hypoxemic at admission, mortality was 4.2%. Pneumonia cases differed from the controls regarding clinical signs and symptoms but not in terms of past medical history. Multivariate analysis of nasal swab findings disclosed that *S*. *pneumoniae* (adjusted odds ratio [aOR] = 3.4, 95% confidence interval [95% CI]: 1.6–7.0), human metapneumovirus (aOR = 17.2, 95% CI: 2.0–151.4), respiratory syncytial virus [RSV] (aOR = 7.4, 95% CI: 2.3–23.3), and influenza A virus (aOR = 10.7, 95% CI: 1.0–112.2) were associated with pneumonia, independently of patient age, gender, period, and other pathogens. Distribution of *S*. *pneumoniae* and RSV differed by season with higher rates of *S*. *pneumoniae* in January-June and of RSV in July-September. Pneumococcal serotypes 1 and 5 were more frequent in pneumonia cases than in the controls (*P* = 0.009, and *P* = 0.04, respectively).

**Conclusions:**

In this non-PCV population from Mali, pneumonia in children was mainly attributed to *S*. *pneumoniae*, RSV, human metapneumovirus, and influenza A virus. Increased pneumococcal conjugate vaccine coverage in children could significantly reduce the burden of pneumonia in sub-Saharan African countries.

## Introduction

Pneumonia is the leading cause of child mortality from infectious diseases, accounting for an estimated 1 million deaths annually, and mainly affecting children in developing countries [1,2]. Mortality attributed to pneumonia has decreased since 2000, but remains a major public health concern [3,4]. The main known causative pathogens reported are *Streptococcus pneumoniae*, *Haemophilus influenzae* type B, and respiratory syncytial virus (RSV) [5]. However, their distribution varies by season and location. Data on the etiology and epidemiology of pneumonia in children in developing countries are still insufficient, particularly in sub-Saharan Africa [6].

Mali is one of the poorest countries in the world, with a under-five year mortality rate of 123 per 1,000 live births in 2013 according to the UN Inter-agency Group for Child Mortality Estimation. It has been estimated that among its 14.9 million inhabitants, each year more than 900,000 pneumonia episodes occur in children under 5 years of age, leading to almost 8,000 deaths annually [7]. A previous descriptive study reported that pneumonia was the most frequent cause of admission, representing 18% of total hospital admissions [8]. However, detailed information was not available on clinical presentation and on the etiology of suspected pneumonia cases [8]. Moreover, no control group was included. The presence of a control group of children without pneumonia would allow better interpretation of microbiological findings, particularly with nasal sampling [9]. In March 2011, Mali included pneumococcal vaccination (PCV13) in a routine immunization program; but vaccine coverage is still low [10].

The study objective was to assess the etiology and factors associated with community-acquired pneumonia in hospitalized children in Mali.

## Materials and Methods

### Setting and Participants

A prospective multicenter case-control study, based on the *Global Approach to Biological Research*, *Infectious diseases and Epidemics in Low-income countries* (GABRIEL) network [11], was implemented in the Pediatric department of Gabriel Touré University Hospital at Bamako, Mali, between July 2011 and December 2012. This multicenter study is ongoing, it will include ten study sites, located in 9 countries over 3 continents (Brazil, Cambodia, China, Haiti, India, Madagascar, Mali, Mongolia, and Paraguay). The study protocol has been described in detail elsewhere [12], and pooled results will be analyzed later.

The Gabriel Touré University Hospital is a 447-bed tertiary-care general hospital located in Bamako. It is a primary care hospital for people living in Bamako and a national reference centre for other patients. Various medical and surgical specialities, including pediatrics, are located in the hospital. The pediatrics department, with a capacity of 150 beds, includes a general pediatrics unit and a neonatal/emergency unit. It receives sick children for primary care and severe cases referred from other healthcare settings. On average, 50,000 consultations and 10,000 hospital admissions occur in the pediatrics department annually. Acute respiratory infections represent 34% of admissions in children, and 15% of child hospitalizations.

### Case Definition and Enrollment

Pneumonia cases were hospitalized children who fulfilled the following criteria:

- Cough and/or dyspnea, and
- Tachypnea, as characterized by the World Health Organization (WHO) in children between 2 and 12 months of age: breathing rate ≥50 cycles per minute; in children between 12 and 59 months of age: breathing rate ≥40 cycles per minute) [13], and
- Absence of wheezing at auscultation, and
- First symptoms appearing within the last 14 days, and
- Radiological confirmation of pneumonia as per WHO guidelines [14].

The exclusion criterions for cases were presence of wheezing at auscultation, or minors whose parents or legal guardian declined to sign the informed consent statement. Controls were patients hospitalized for surgery or in a routine outpatient practice environment, aged between 2 and 59 months, without any symptoms suggestive of respiratory illness; suspicion of infection of other site was not an exclusion criteria. Cases and controls were matched for age (±1 year) and calendar date of hospital admission (±1 month) to take seasonality into account. Thus, 61% of patients were recruited during the rainy season (May to October) while 39% were recruited during the dry season (November to April).

### Biological Samples

Samples were collected in the first 48 hours of patient hospitalization. Nasal swabs were taken from all pneumonia cases and controls. To document antibiotic usage history, urine was sampled from cases for broad spectrum antibiotic detection. Whole blood and pleural effusions (in applicable cases) were collected from cases only. Whole blood was distributed in Ethylenediaminetetraacetic acid (EDTA) vials for preservation and in dry tubes for serum specimens. EDTA-whole blood samples allowed complete blood count, blood culture and real-time multiplex polymerase chain reaction (PCR) assay for the identification of *Staphylococcus aureus*, *Streptococcus pneumoniae* and *Haemophilus influenzae* type b. C reactive protein (CRP) and procalcitonin (PCT) were measured quantitatively in serum.

Respiratory specimens from nasal swabs and pleural effusions were characterized by FTD respiratory pathogens 21 plus (Fast-track Diagnostics, Luxembourg) based on RT-PCR which included a panel of 19 viruses and 5 bacteria. The pathogens identified were: influenza A, influenza A(H1/N1), influenza B, coronavirus 229E, coronavirus OC43, coronavirus NL63, coronavirus HKU1, parainfluenza virus 1, parainfluenza virus 2, parainfluenza virus 3, parainfluenza virus 4, human metapneumoviruses A and B, rhinovirus, RSVs A and B, adenovirus, enterovirus, parechovirus, bocavirus, *Mycoplasma pneumoniae*, *Chlamydia pneumoniae*, *S*. *aureus*, *S*. *pneumoniae*, and *H*. *influenzae* type b. *S*. *pneumoniae*-positive specimens were serotyped by RT-PCR that detects the 29 main *S*. *pneumoniae* serotypes: 1, 3, 4, 5, 6A/B, 7C, 7F, 8, 9V, 10A, 11A, 12F, 14, 15A, 15B/C, 16F, 17F, Sg18C, 19A, 19F, 20, 22F, 23F, 31, 33F, 34, 35B, 35F, 38 and Lyt A.

### Statistical Analysis

Demographic characteristics, underlying diseases, medical history, clinical examination at enrollment, radiological findings, vaccinations, and outcome were recorded prospectively for each patient on a standardized form.

Continuous variables were described as median and interquartile range (IQR), categorical variables as number and percentage. Pneumonia cases we compared to the controls by the Mann-Whitney U test for continuous covariates, and by the Chi^2^ test for categorical variables. Univariate and multivariate logistic regression analyses were performed to assess factors associated with pneumonia. Multivariate analyses were adjusted for gender, age, period per quarter, and other pathogens significantly associated with increased risk of pneumonia. Concordance between serotypes detected in nasopharyngeal samples and blood was tested by Kendall rank correlation.

Crude population attributable fraction (PAF) was calculated after univariate logistic regression analysis to quantify the contribution of the each microorganism to pneumonia occurrence. Conceptuality, PAF permits the estimation of the proportional reduction in pneumonia occurrence that would occur if the pathogen was absent (alternative ideal scenario). The formula was the following:

$$PAF=\frac{{Pr}_{c}\times \left(OR-1\right)}{1+{Pr}_{c}\times \left(OR-1\right)}$$

$$\text{With}:\;{Pr}_{c}=\mathrm{Prevalence of microorganim detection in controls},\;\text{O}R=\mathrm{crude odds ratio}.$$

No patient was excluded because of missing data. *P*<0.05 was considered significant; all tests were bilateral, and statistical analysis was conducted with Stata version 13.0 (Stata Corp.). With at least 100 cases and 100 controls, analyses had 80% power to detect odds ratios (OR)≥3 with ≥20% prevalence of control exposure, whatever the prevalence of case exposure.

### Ethics

The study protocol, informed consent statement, clinical research form, any amendments and all other study documents were submitted to and approved by the institutional ethics committee (*Comité National d'Ethique pour la Santé et les Sciences de la Vie*). Written informed consent was obtained from all participants.

## Results

### Overall Population

Among the 119 enrolled cases, 118 conformed with the inclusion criteria and were all sampled. One patient without radiological confirmation was excluded from the analysis. Among the 100 included controls, 2 were excluded because of missing data.

Overall, 216 patient, accounting for 118 (54.6%) pneumonia cases and 98 (45.4%) controls, were analyzed. Among them, 93 (44.1%) were female. The male/female gender ratio was 0.79, and median age was 11 months (IQR: 5–55 months).

### Description of Pneumonia Cases

Among the 118 patients with pneumonia, 16 (13.6%) were referred from another health center; median length of hospital stay was 7 days (IQR: 6–10 days) for a total of 909 days of hospitalization (Table 1). The median time period between first symptoms and hospitalization was 6 days (IQR: 4–7 days).

**Table 1:** Characteristics of children pneumonia cases and controls, Mali, N = 216.

Characteristics^a^	Pneumonia cases (n = 118)	Controls (n = 98)	*P*
**Demographics at admission**			
Gender, male	57 (48.3)	36 (38.7)	0.16
Age, months, median (IQR)	12 (5–26)	11 (5–23)	0.24
Height, cm, median (IQR)	73.5 (65–88)	71 (64–79)	0.04
Weight, kg, median (IQR)	7.6 (6–10)	7 (5.5–9.3)	0.13
Body mass index, median (IQR)	13.9 (12.6–15.2)	14.2 (12.9–15.5)	0.47
Arm circumference, cm, median (IQR)	13 (12–14)	12.5 (11–14)	0.01
Weight-for-height Z-score ≤2 SD	52 (44.1)	41 (44.1)	0.99
Weight-for-height Z-score ≤3 SD	32 (27.1)	22 (23.7)	0.57
Medical history			
Heart disease	4 (3.4)	0 (0)	0.07
HIV-positive	3 (2.5)	0 (0)	0.12
Contracted a common cold/pharyngitis^b^	61 (31.7)	0 (0)	<0.001
Contracted ILI^b^	18 (15.2)	0 (0)	<0.001
Pneumococcal conjugate vaccine	0 (0)	0 (0)	-
DPT-Hep. B-Hib vaccine, 1 dose	102 (86.4)	80 (86.0)	0.93
DPT-Hep. B-Hib vaccine, 3 dose	81 (68.6)	69 (74.2)	0.38
Influenza vaccine	1 (0.8)	0 (0)	0.37
**Vital signs at admission**			
Temperature, °C, median (IQR)	38.6 (37.8–39.4)	37 (36.8–37.4)	<0.001
Breathing rate, cycles/min, median (IQR)	56 (48–67)	31 (29–35)	<0.001
Cardiac rate, cycles/min, median (IQR)	151 (132–167)	122 (112–132)	<0.001
Oxygen saturation, %, median (IQR)	94.5 (87–96)	98 (98–99.5)	<0.001
**Clinical signs/symptoms at admission**			
Dyspnea	116 (98.3)	0 (0)	<0.001
Lower chest indrawing	116 (98.3)	0 (0)	<0.001
Cough	114 (96.6)	0 (0)	<0.001
Pulmonary crackles	102 (86.4)	0 (0)	<0.001
Ronchi	34 (28.8)	0 (0)	<0.001
Rhinopharyngitis	32 (27.1)	0 (0)	<0.001
Prostration or lethargy	23 (19.5)	0 (0)	<0.001
Inability to drink	15 (12.7)	0 (0)	<0.001
Diarrhea	12 (10.2)	20 (21.5)	0.02
Cyanosis	11 (9.3)	0 (0)	0.002
Vomiting	9 (7.6)	13 (14.0)	0.13
Convulsions	6 (5.1)	2 (2.1)	0.27
Conjunctivitis	6 (5.1)	1 (1.1)	0.11
Diminished breath sounds	5 (4.2)	0 (0)	0.04
Dullness to percussion	4 (3.4)	0 (0)	0.07
Otitis	3 (2.5)	1 (1.1)	0.44
Rasping	3 (2.5)	0 (0)	0.12
Skin rash	0 (0)	2 (2.1)	0.11

Abbreviations: IQR, interquartile range; SD, standard deviation; DPT-Hep. B-Hib, Diphtheria, tetanus, Pertussis, hepatitis B, *Haemophilus influenza* type b; HIV, human immunodeficiency virus; ILI, influenza-like illness

^a^Data are N (%), unless specified otherwise.

^b^within 2 weeks.

Four (3.9%) pneumonia cases presented a ventricular septal defect. None had previous tuberculosis or underlying lung disease. Among cases with pulmonary crackles, 36 (35.3%) were unilateral and 66 (64.7%) were bilateral. Other signs or medical history of pneumonia cases included sickle-cell anaemia (*n* = 1), dehydration (*n* = 1), epilepsy (*n* = 1), goiter (*n* = 1), edema (*n* = 1), pallor (*n* = 1), and trisomy (*n* = 3).

Among the 47 patients who took antibiotics before hospitalization, 17 (14.4%) received Amoxicillin, 8 (5.6%) Ceftriaxone, 4 (2.8%) Amoxicillin/Clavulanic acid, and 3 (2.1%) Cotrimoxazole. Among the 63 cases tested for antibiotic detection testing in urine, 22 (65.1%) were positive; in this population, sensitivity of declared antibiotic use compared with urine detection was low (sensitivity = 51.2%, 95% confidence interval: 35.1–67.1%).

Median CRP level at admission was 21 mg/l (IQR: 6–63, mean: 56.6 mg/l), median PCT level was 4.6 ng/ml (IQR: 0.4–26.4, mean: 31.5 ng/ml). Median CRP and PCT levels were higher in hypoxemic compared with non-hypoxemic pneumonia cases (*P* = 0.03 and *P* = 0.007, respectively).

Median white blood cell count was 15,750*10^9^cells/l (IQR: 10,000–25,700, mean: 20,600*10^9^cells/l), and median neutrophil percentage was 52.5% (IQR: 31–68, mean: 50.3%). X-rays showed generalized, dense, homogeneous opacification in 24 (20.3%) patients, interstitial syndrome in 24 (20.5%), and alveolar infiltrate in 65 (55.1%), without significant differences according to the pattern of infection (viral, bacterial or mixed). No patient had abscess or pneumothorax, but 4 (3.4%) had pleural effusion.

During hospital stay, pneumonia cases received the following antibiotics: Amoxicillin (*n* = 111, 94.1%), Ceftriaxone (*n* = 3, 2.6%), Amoxicillin/Clavulanic acid (*n* = 3, 2.5%), Ciprofloxacin (*n* = 1, 0.8%), and Vancomycin (*n* = 2, 1.6%). Fifty-one (43.2%) received oxygen for a median length of 2 days (IQR: 1–3 days), and 3 (2.5%) had blood transfusions. Three patients with pleural effusions were tested for microbial detection. Five patients died (lethality: 4.2%); death was directly related to pneumonia in 4 patients (Table 2).

**Table 2:** Description of deceased patients with pneumonia, N = 5.

ID	Gender	Age	Signs	Respiratory rate	Temperature	Oxygen saturation	Delay from admission to death	Treatments	Blood, molecular detection or culture	Nasal aspirate Microbiology, molecular detection	Cause of death
#1	F	4 years	Dyspnea, lower chest indrawing	77 cycles/min	38.4°C	86%	10 hours	Amoxicillin, paracetamol, oxygen	*S*. *pneumoniae* serotype 3^a^	*S*. *aureus*, *H*. *influenza*, Parainfluenzae 2	Severe pneumonia
#2	F	3 months	Dyspnea, lower chest indrawing	47 cycles/min	39 .7°C	89%	2 days	Amoxicillin, paracetamol, oxygen	Negative	Coronavirus HKU1	Severe pneumonia
#3	F	2 years	Dyspnea, lower chest indrawing, dehydration, pallor	82 cycles/min	40.1°C	89%	12 hours	Amoxicillin, gentamycin, rehydration, paracetamol, oxygen	*S*. *pneumoniae* serotype 35F^a^	*S*. *pneumonia*e serotypes 19A, 19F, 22F, 35F *S*. *aureus*, parainfluenzae 3, enterovirus	Severe, acute malnutrition
#4	M	8 months	Dyspnea, lower chest indrawing,	62 cycles/min	38.8°C	80%	12 hours	Amoxicillin, gentamycin, parecetamol, oxygen	Coagulase-negative staphylococci^b^	*S*. *pneumoniae* serotypes 6AB, 15BC, *H*. *influenza*, RSV	Severe pneumonia
#5	F	13 months	Dyspnea, lower chest indrawing, pallor	60 cycles/min	38.7°C	68%	3 days	Amoxicillin, paracetamol, oxygen	Coagulase-negative staphylococcib	S. pneumoniae serotype 7C, Bocavirus	Pneumonia with moderate, acute malnutrition

Abbreviations: F, female; M, male; RSV, respiratory syncytial virus.

^a^ By PCR

^b^ Blood culture

### Comparison of Characteristics of Pneumonia Cases and Controls

Table 1 compares the clinical signs and symptoms at admission between cases and controls, underlying conditions and biological findings. Pneumonia cases differed from controls regarding clinical signs and symptoms as well as vital signs at admission, but not in terms of demographic factors or past medical history. Pneumonia cases were more frequently hypoxemic (defined as oxygen saturation<90%) at admission than the controls (30.5% vs. 0%, *P*<0.001). PCV coverage was zero in both groups.

### Microbiological Findings

**Fig 1:**
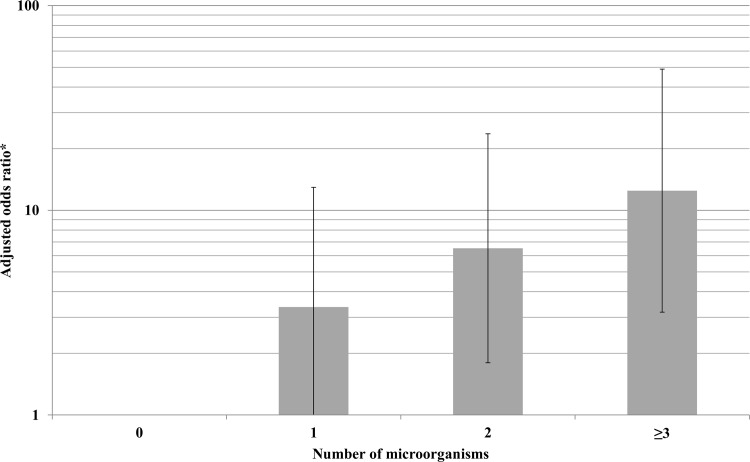
Relative risk (95% confidence interval) of pneumonia in children according to number of microorganisms from nasal swab, Mali, N = 216. ^a^After multivariate logistic regression, adjusted for gender, age, and period per quarter. ^b^Logarithmic scale.

**Fig 2:**
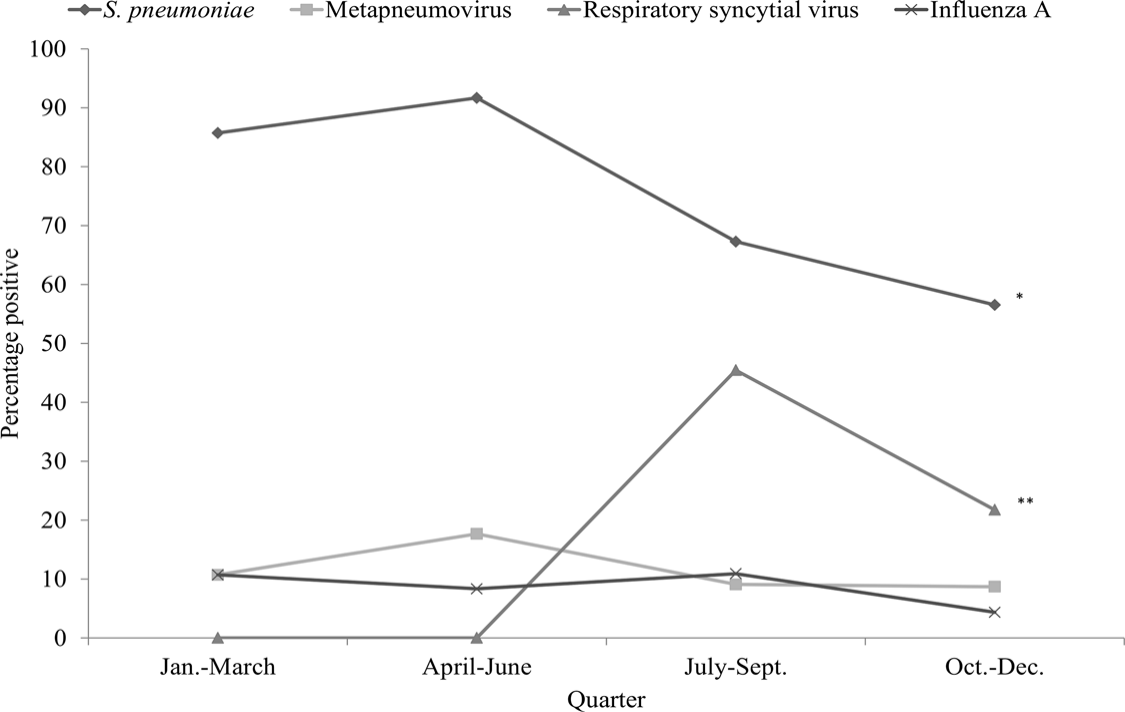
Temporal trends of pneumonia etiology in childhood cases, Mali, N = 118. *P < .05, **P < .001.

At least 1 microorganism was detected on nasal swabs in 96.6% of cases and 82.3% of controls (crude OR = 6.4, 95% confidence interval [95% CI]: 2.1–19.7, *P*<0.001). Overall, 78.8% of cases and 54.2% of controls were co-infected or co-colonized (crude OR = 3.3, 95% CI: 1.8–6.0, *P*<0.001). Co-detection on nasal swab of *S*. *pneumoniae* and RSV was more frequent in cases than in controls (respectively, 15.2% [N = 18] vs. 2.0% [N = 2], *P* = 0.001). Co-detection of *S*. *pneumoniae* and rhinovirus was not different in cases and controls (respectively, 16.1% [N = 19] vs. 12.2% [N = 12], *P* = 0.42; co-detection RSV and rhinovirus was not different between cases and controls (respectively, 5.9% [N = 7] vs. 3.1% [N = 3], *P* = 0.32). A dose-response relationship was apparent between the number of microorganisms found in nasal swabs and the risk of being a case (Fig 1). Distribution of *S*. *pneumoniae* and RSV differed by season with higher rates of *S*. *pneumoniae* in January-June and of RSV in July-September (Fig 2).

Univariate analysis revealed that *S*. *pneumoniae*, human metapneumovirus, RSV, and influenza A virus detection in nasal swabs were significantly associated with pneumonia in Mali (Table 3). Multivariate analysis reinforced linkage of these pathogens with pneumonia, independently of patient age, gender, period per quarter and the presence of other pathogens significantly coupled with increased risk of pneumonia (Table 3). PAF was the highest for *S*. *pneumoniae* (PAF = 46%, 95% CI: 30–59%). Contribution of human metapneumovirus, RSV, and influenza A were lower, with PAFs of 9% (95% CI: 7–11%), 21% (95% CI: 16–25%) and, 8% (95% CI: 6–10%), respectively.

**Table 3:** Microbiological findings of children pneumonia cases and controls, nasal swabs, Mali, N = 216.

Microorganisms	Pneumonia cases (n = 118)	Controls (n = 98)	*P*	Crude odds ratio (95% CI)	Adjusted odds ratio^a^ (95% CI)
**Bacteria**					
*Streptococcus pneumoniae*	85 (72.0)	47 (48.0)	<0.001	2.8 (1.6–4.9)	3.4 (1.6–7.0)
*Staphylococcus aureus*	23 (19.5)	19 (19.4)	0.98	1.0 (0.5–2.0)	-
*Haemophilus influenza*	8 (6.8)	6 (6.2)	0.84	1.1 (0.4–3.3)	-
*Mycoplasma spp*.	0 (0)	1 (1.0)	0.27	NE	-
*Chlamydia spp*.	0 (0)	0 (0)	-	NE	-
**Viruses**					
Human metapneumovirus	12 (10.2)	1 (1.0)	0.005	11.0 (1.4–86.0)	17.2 (2.0–151.4)
Coronavirus NL63	1 (0.8)	4 (4.1)	0.12	0.2 (0.02–1.8)	-
Coronavirus 229E	2 (1.7)	0 (0)	0.19	NE	-
Coronavirus OC43	2 (1.7)	4 (4.1)	0.29	0.4 (0.07–2.3)	-
Coronavirus HKU 1	6 (5.1)	2 (2.0)	0.24	2.6 (0.5–13.0)	-
Adenovirus	11 (9.3)	6 (6.1)	0.39	1.6 (0.6–4.4)	-
Enterovirus	12 (10.2)	14 (14.3)	0.35	0.7 (0.3–1.5)	-
Parechovirus	0 (0)	0 (0)	-	NE	-
Rhinovirus	27 (22.9)	24 (24.5)	0.78	0.9 (0.5–1.7)	-
Respiratory syncytial virus	30 (25.4)	6 (6.1)	<0.001	5.2 (2.1–13.2)	7.4 (2.3–23.3)
Parainfluenzae 1	5 (4.2)	0 (0)	0.04	NE	-
Parainfluenzae 2	1 (0.8)	0 (0)	0.36	NE	-
Parainfluenzae 3	5 (4.2)	3 (3.1)	0.65	2.0 (0.4–10.6)	-
Parainfluenzae 4	2 (1.7)	4 (4.1)	0.29	0.4 (0.07–2.1)	-
Influenza A	11 (9.3)	1 (1.0)	0.008	10.0 (1.3–78.7)	10.7 (1.0–112.2)
Infuenza B	0 (0)	0 (0)	-	NE	-
Influenza A(H1N1)	3 (2.5)	0 (0)	0.11	NE	-
Bocavirus	13 (11.0)	12 (12.2)	0.78	0.9 (0.4–2.0)	-

Abbreviations: NE, non estimable.

^a^ After multivariate logistic regression, adjusted for the presence of other pathogens, gender, age, and period per quarter.

**Fig 3:**
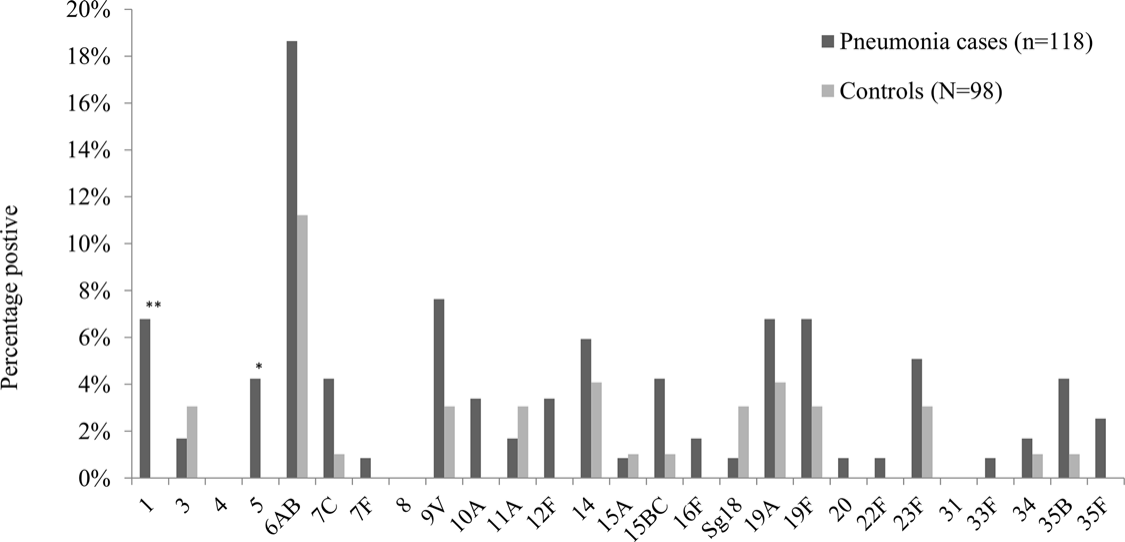
*Streptococcus pneumoniae* serotypes in nasal aspirates from pneumonia cases and controls, Mali, N = 216. *P < .05, **P < .001.

Fig 3 reports the distribution of pneumococcal serotypes detected in nasal swabs from cases and controls. The most prevalent serotype in pneumonia cases and controls was serotype 6A/B (18.6% vs. 11.2%, *P* = 0.13). Serotypes 1 and 5 were more frequent in pneumonia cases than in the controls: 6.8% vs. 0%, *P* = 0.009, and 4.2% vs. 0%, *P* = 0.04, respectively.

In pneumonia cases, *S*. *pneumoniae* was positive in 16 (13.6%) patients, *S*. *aureus* in 6 (5.1%) patients and *H*. *influenza* in 5 (4.2%) patients by PCR blood sample detection. Most patients with *S*. *pneumoniae* detection by PCR had also *S*. *pneumoniae* nasal carriage (93.5%, 15/16), while only 17.6% (15/85) of patients with *S*. *pneumoniae* nasal carriage had positive detection by PCR (*P* = 0.04). Concordance of serotype 1 detected in nasal swabs and blood in pneumonia cases was high (κ = 0.70, *P*<0.001). Coronavirus 63 was identified from pleural effusion in 1 patient. Microbiological findings, including *S*. *pneumoniae* serotypes distribution, from PCR nasal swab or blood sample were not different in pneumonia cases according to the result of urine antibiotic testing (data not shown). Blood culture was positive in 36 (30.5%) pneumonia cases; most microorganisms were probably related to contamination of samples. The following bacteria were detected: coagulase-negative staphyloccoci (*n* = 26), *Salmonella spp*. (*n* = 3), Gram-positive bacilli (*n* = 2), *Acinetobacter baumannii* (*n* = 1), *Aerococcus viridans* (*n* = 1), *Enterococcus faecium* (*n* = 1), *Granulicatella elegans* (*n* = 1), and *Staphylococcus aureus* (*n* = 1).

## Discussion

The primary objective of this prospective case-control study was to assess the etiology and factors associated with community-acquired pneumonia in hospitalized children in Mali. In this non-PCV population, we observed that *S*. *pneumoniae*, human metapneumovirus, RSV, and influenza A were the main microbial agents associated with pneumonia among children in Mali, independently of patient age, gender, period, and other pathogens. *H*. *influenzae* was not associated with pneumonia, but the vaccination rate against this bacteria, at least for the first dose, was above 85% in both cases and controls. In addition, while most patients with *S*. *pneumoniae* by PCR in blood had also nasal carriage, frequently with the same serotype, positive *S*. *pneumoniae* test from nasal sample was not highly predictive of *S*. *pneumoniae* detection in blood, meaning that the bacteria was frequently not the cause of pneumonia.

Few studies have assessed the etiology of pneumonia in sub-Saharan Africa. Howie *et al*. recently investigated pneumonia etiology in children in Gambia, observing that S. *pneumoniae* was the leading cause with a high rate of microbial agent co-detection [15]. Conversely, they found that viral pneumonia was not predominant whereas we observed that 3 viruses, namely human metapneumovirus, RSV, and influenza A virus, were associated with pneumonia in Mali. Major differences between the 2 studies were sample type (lung aspirates *vs*. nasal swabs in our investigation) and the lack of a comparative group in the study by Howie *et al*. which did not permit comparison of microbial prevalence in pneumonia patients and healthy subjects [15]. In a Western Kenya case-control study, *S*. *pneumoniae*, RSV, and influenza A virus were the predominant causes of pneumonia in children [16]. We noted almost similar results in Mali. Thus, *S*. *pneumoniae* and RSV could still be considered as the primary causes of pneumonia in several sub-Sharan African countries.

All patients who died had severe pneumonia, according to WHO criteria, with significant dyspnea, very marked chest indrawing and severe hypoxia. Two patients had confirmed pneumococcal pneumonia. Two patients had acute malnutrition linked with pneumonia. In 2 cases, death occurred within 24 hours of admission. Despite active treatment with antibiotics and oxygen, the management of severe respiratory distress is often difficult in this context because the hospital does not have assisted ventilatory support. Mortality was higher than in a recent birth cohort in South Africa [17], but was similar to what was reported previously in other developing countries [18,19].

Some invasive serotypes were detected selectively in pneumonia cases but not in the controls (i.e., serotypes 1 and 5 were associated with the risk of pneumonia). A previous study of serotypes involved in invasive pneumococcal disease in Mali found that serotype 5 was the most prevalent (54%) [20]. It was also linked with pneumonia in our series. In other sub-Saharan African countries, serotype 1 was described as the most prevalent serotype of invasive pneumococcal disease [21]. It was the second serotype significantly associated with pneumonia in children in Mali. Thus, the introduction of PCV in routine immunization programs in Mali would substantially reduce pneumonia caused by *S*. *pneumoniae* because most serotypes eliciting pneumonia would be covered by the vaccine [22]. In addition, pneumococcal pneumonia seasonality was similar to that observed previously in Burkina Faso, a neighbouring country, in 2007–2011 with higher incidence during the dry season between January and May [23].

Interestingly, among the 4 microbial agents associated with pneumonia, 3 were viruses: human metapneumovirus, RSV, and influenza A virus. Self *et al*. recently observed, in a pneumonia cases-control study implemented in hospitals of Utah, that detection respiratory syncytial virus, human metapneumovirus and influenza from nasopharyngeal or oropharyngeal sample of patients with pneumonia probably indicates an etiologic role [24].

We observed similar results in a different context. Despite the dearth of vaccination against pneumococcus in our population, viral pneumonia is a major segment of the pneumonia burden. Treatment of these infections is often problematic because the empirical use of antibiotics or antivirals is not consensual [25]. Vaccine development, particularly against RSV, is warranted to prevent pneumonia in children. Moreover, the impact of influenza vaccine policies in developing countries should be evaluated because this virus often causes pneumonia [26].

Rhinovirus was detected in almost 25% of pneumonia cases of our series, but the detection rate was not different between cases and controls. However, this observation does formally excludes that rhinovirus could play a role in the etiology of pneumonia. Jain et al. recently observed that rhinovirus was detected in 27% of U.S. children with community-pneumonia requiring hospitalization [27]; this large study had however no control group, it was also not possible to estimate the prevalence of respiratory detection in children without pneumonia. Growing evidence suggests the role of rhinovirus in the etiology of viral pneumonia [28] or related to the risk of secondary bacterial pneumonia [29]. Other viruses, such as bocavirus or adenovirus were also equally prevalent in cases and controls. Again, this finding does not exclude completely their implication in pneumonia etiology in children from Mali. However these viruses were poorly described as causative agents of pneumonia in children from other sub-Saharan African countries.

Several microorganisms have been detected in most pneumonia cases but also in the controls. The clinical significance of microbial detection by PCR is problematic at the individual level because most detected pathogens are not causes of pneumonia. However, number of pathogens is predictive of the risk of pneumonia, suggesting that simple quantification of species detected would permit the evaluation of pneumonia risk. At the population level, nasal samples are interesting because the prevalence of carriage in the controls is considered.

The main strengths of our study were its prospective design with the inclusion of controls that permitted us to assess pneumonia etiologies while taking into account the prevalence of pathogen detection in non-infected patients. Ours is the first case-control investigation of pneumonia etiology in children from Mali (S1 STROBE checklist). The results should serve to better manage pneumonia not only in these children but also in those from neighboring countries. Data quality was enhanced by centralized microbiological analysis in the Emerging Pathogens Laboratory (Lyon, France). In addition, this analysis should serve to better focus multicentric pneumonia studies that will permit us to assess the global etiologies of pneumonia and to compare etiologic agents between countries and continents.

Some limitations should be underlined. First, microbiological diagnosis of pneumonia is difficult due to the lack of single reliable test. Then, at the individual level, it is difficult to establish if a positive nasal swab denotes etiology or nasopharyngeal colonization; particularly for bacterial agents such as *S*. *pneumoniae* because of the high asymptomatic carriage. However, at the population level, addition of a control group permits to evaluate and control for the prevalence of carriage in asymptomatic children. In a pneumonia etiology study, it would be interesting to correlate the threshold cycle (Ct) values obtained from RT-PCR with lower airway specimens to establish more precisely the relationship between positive nasal swab and etiology of pneumonia, according to the microorganism [30]. Second, because the study was implemented in one hospital in Mali, external validity might be limited. However, Gabriel Touré University Hospital is a reference center in the country, and then source population is not limited to the city of Bamako. Third, study power was confined to detecting some linkages (i.e. serotypes associated with the risk of pneumonia).

## Conclusion

Community-acquired pneumonia was mainly attributable to *S*. *pneumoniae*, human metapneumovirus, RSV or influenza A among children in Mali. Increased pneumococcal conjugate vaccine coverage in children would significantly reduce the burden of pneumonia in this country. The addition of a control group to assess etiologies of pneumonia in children is critical to properly interpret the microbiological results of diagnostic testing with high sensitivity.

## Supporting Information

S1 STROBE Checklist (DOC)

S1 Table Characteristics of children pneumonia cases and controls, Mali, N = 216. (XLS)

S2 Table Microbiological findings of children pneumonia cases and controls, nasal swabs, Mali, N = 216. (XLS)

## Abbreviations

CRP: C-reactive protein
FTD: Fast-track Diagnostics
IQR: interquartile range
OR: odds ratio
PCV: pneumococcal conjugate vaccination
PCT: procalcitonin
RSV: respiratory syncytial virus
RT-PCR: real-time multiplex polymerase chain reaction
WHO: World Health Organization
